# Enhanced Pure Pursuit Path Tracking Algorithm for Mobile Robots Optimized by NSGA-II with High-Precision GNSS Navigation

**DOI:** 10.3390/s25030745

**Published:** 2025-01-26

**Authors:** Xiongwen Jiang, Taiga Kuroiwa, Yu Cao, Linfeng Sun, Haohao Zhang, Takahiro Kawaguchi, Seiji Hashimoto

**Affiliations:** 1 Division of Electronics and Informatics, Gunma University, Kiryu 376-0052, Japan; jxw.chinese.japan@gmail.com (X.J.); t190d509@gunma-u.ac.jp (T.K.); felicityslf@outlook.com (L.S.); h-zhang@gunma-u.ac.jp (H.Z.); kawaguchi@gunma-u.ac.jp (T.K.); 2 Faculty of Informatics, Aramaki Campus, 4-2 Aramaki-Machi, Maebashi-shi 371-0044, Japan; caoyu@gunma-u.ac.jp

**Keywords:** GNSS, path tracking, localization, navigation

## Abstract

With the rapid development of automation and intelligent technology, mobile robots have shown wide application potential in many fields, and accurate navigation systems are the key to robots completing tasks. This paper proposes an enhanced pure pursuit path tracking algorithm for mobile robots, which is optimized using NSGA-II, with high-precision GNSS navigation for accurate positioning. The improved algorithm considers the dynamic characteristics and real–world operating conditions of the robot, optimizing steering decisions to enhance path tracking accuracy. Experimental results demonstrate the effectiveness of the algorithm: with a look–ahead distance of 0.5 and a maximum linear velocity of 3, the average absolute pose error (APE) is reduced by 14.63%, while a velocity of 4 reduces the APE by 55.94%. The enhanced algorithm significantly reduces path deviation and improves navigation performance.

## 1. Introduction

### 1.1. Mobile Robotics Applications

Mobile robots have found widespread applications in fields such as unmanned aerial vehicles (UAVs), autonomous vehicles, industrial robots, and service robots [1]. In these domains, the performance of navigation systems is critical for ensuring the efficiency and safety of task execution. UAVs are extensively utilized in scenarios such as logistics, agriculture, disaster rescue, and environmental monitoring [2]. Their navigation systems must integrate high−precision localization, obstacle avoidance capabilities, and real–time path planning [3].

Autonomous vehicles represent a significant application of mobile robot technology, with navigation systems that must address highly complex requirements, including precise environmental perception, high-definition mapping, and centimeter-level localization accuracy [4]. These vehicles leverage a combination of sensors, such as GNSS, LiDAR, and cameras, to perceive traffic environments in real time and make informed decisions, enabling efficient and safe driving [5]. By optimizing paths and planning behaviors in real time, these systems can dynamically adjust driving strategies based on traffic conditions, ensuring operational safety in both intricate urban settings and high-speed highways [6].

### 1.2. GNSS−Based Localization and Navigation

Global Navigation Satellite System (GNSS) provides a robust tool for enabling the precise positioning of mobile robots in expansive outdoor environments [7]. By receiving signals from Earth–orbiting satellites, GNSS allows robots to determine their position within a global coordinate system [8]. This technology has found widespread applications in fields such as agriculture [9], drone development [10], and logistics, significantly contributing to automation and efficiency in these domains. However, GNSS technology faces several limitations in practical applications, including multipath effects [11], signal obstruction, positioning errors, and a strong dependency on satellite signals [12], which constrains its applicability in complex environments.

One significant limitation of GNSS is the multipath effect, particularly in urban environments [11]. In such settings, tall buildings, bridges, and other reflective surfaces can cause GNSS signals to be reflected or refracted, leading to substantial positioning errors. Moreover, in scenarios such as forests or canyons, GNSS signals are often attenuated or blocked, resulting in degraded system stability and accuracy [13]. These challenges severely undermine the reliability of GNSS in complex operational contexts.

To address these issues, researchers have proposed various improvement methods. For instance, ref. [14] proposed an enhanced GPS sensor model, which was developed to improve the positioning accuracy of mobile robots in urban environments, effectively mitigating the impact of multipath effects. Additionally, robust optimization algorithms have been designed to alleviate the impact of multipath interference, such as signal filtering techniques based on environmental modeling [15]. In open environments, GNSS solutions employing real-time kinematic (RTK) techniques have demonstrated substantial accuracy improvements [16].

Multi-sensor fusion [17] has become a crucial approach for addressing GNSS limitations, significantly improving navigation system accuracy and robustness. GPS provides global positioning, while IMU estimates motion states through acceleration and angular velocity measurements. Their integration allows IMU to mitigate GPS instability or signal loss and correct cumulative errors. However, in challenging environments like urban canyons or tunnels, GPS obstruction and IMU drift pose challenges. To address these, Kalman filtering is commonly employed, effectively reducing noise and enhancing system stability [18]. The fusion method’s success hinges on accurate sensor models and efficient noise management.

### 1.3. Comparative Analysis of Path Tracing Algorithms

Path tracking algorithms are crucial for autonomous systems to accurately follow predefined trajectories. Numerous algorithms have been developed, each offering specific advantages and limitations in various application scenarios. Pure Pursuit is widely recognized for its simplicity and ease of implementation [19]. It performs well at low speeds, but exhibits significant accuracy degradation at high speeds, particularly in sharp turns or complex paths. Despite these limitations, Pure Pursuit remains a preferred choice in foundational research and industrial applications due to its low computational cost and minimal dependence on system modeling.

To address these limitations, extensive research has been conducted by scholars worldwide. Ref. [20] proposed an improved pure pursuit method, with a heuristically selected look–ahead point, enhancing the path tracking accuracy and stability on complex roads. Ref. [21] presented a lateral control system based on an adaptive pure pursuit algorithm, achieving improved path tracking accuracy on curved roads while maintaining performance on low-curvature paths. Ref. [10] proposed an adaptive fuzzy pure pursuit algorithm enhanced with model predictive control (MPC) and whole–body motion control (WBC), significantly improving the stability for autonomous unmanned ground vehicles navigating complex terrains. Ref. [22] proposed an enhanced pure pursuit algorithm for path tracking in multi–UAS systems, utilizing an offline lookup table to optimize goal point selection, ensuring safe separation, complete coverage, and minimal deviation from straight–line trajectories during coverage tasks. Ref. [23] proposed an optimized path tracking algorithm for agricultural machinery by adaptively selecting an optimal goal point based on an evaluation function to minimize lateral and heading errors.

Model Predictive Control (MPC) provides an optimization framework that predicts future states and optimizes control inputs, making it highly adaptable to complex paths and dynamic environments. Ref. [24] proposed an improved MPC controller with fuzzy adaptive weight control for autonomous vehicle path tracking, offering better tracking accuracy, dynamic stability, and smoother steering. Ref. [25] proposed a C-based algorithm that solves the real-time optimization problem, and the system was validated in hardware-in-the-loop simulations. Ref. [26] proposed a combination of a virtual vehicle concept and MPC to address the path-following problem of omnidirectional mobile robots while maintaining velocity constraints. Ref. [27] presented an embedded nonlinear MPC strategy for autonomous vehicles, which is optimized for minimum time using a path-parametric formulation and sequential quadratic programming for real-time control. However, despite these advancements, core challenges persist in MPC, particularly in real-time computation [28], handling nonlinearities effectively, and maintaining robustness under varying environmental conditions and model uncertainties [29]. Future improvements may focus on optimizing computational efficiency, enhancing real-time adaptability, and improving the controller’s performance in unpredictable or highly dynamic environments [30].

Multi sensor fusion techniques integrate data from diverse sensors (e.g., GPS, IMU, LiDAR, and visual sensors) to enhance the robustness and accuracy of path tracking systems [31]. These methods excel in mitigating sensor noise and signal loss, making them particularly suitable for complex and dynamic environments [32]. However, multi sensor fusion involves sophisticated algorithm design and data processing, demands substantial computational resources, and may encounter challenges related to sensor inconsistency [33].

Vision−based tracking has emerged as a promising approach that utilizes visual sensors such as cameras to capture environmental information [34]. This method excels at detecting dynamic obstacles and providing detailed environmental characteristics [35], making it ideal for autonomous vehicles and mobile robots. However, it is highly sensitive to environmental factors such as changes in light occlusions [36]. Additionally, vision−based tracking requires significant processing power, limiting its applicability in cost-constrained platforms [37].

Based on a comprehensive analysis of the advantages and limitations of various path tracking algorithms, we chose to enhance the Pure Pursuit algorithm. Despite its inherent drawbacks, such as reduced accuracy at high speeds and difficulties with sharp turns, the fundamental strengths of Pure Pursuit, i.e., its low computational cost and simplicity, make it a strong foundation for improvement [38]. Our enhancements focus on mitigating these limitations without compromising its core benefits. Our work aims to retain the computational efficiency of Pure Pursuit while improving its performance in high-speed scenarios and on complex paths, thus enhancing its practicality and robustness. The advantages and limitations of path tracking algorithms are shown in Table 1.

### 1.4. Thesis Outline

In Section 2, the measurement models of the robot system are deeply analyzed and verified. First, the rigid body kinematic model is introduced to provide a basis for understanding the dynamics of the robot’s motion in space. Then, the measurement model of the inertial measurement unit (IMU) is explored, where how to use the IMU to obtain the robot’s dynamic information, such as acceleration and angular velocity, is explained.

As shown in Section 3, we designed and developed a mobile robot navigation system based on a high-precision global navigation satellite system (GNSS). The system combines an improved Pure Pursuit path tracking algorithm to improve the robot’s path tracking ability. The theoretical basis of the system design is introduced in detail, including a system overview, GNSS high-precision positioning technology, and the principle and improvement of the pure pursuit tracking algorithm.

## 2. Measurement Model Description

This section provides a comprehensive discussion of the kinematic and sensor models of robots. Initially, the chapter explores the rigid body kinematic model, which details the movement dynamics of the robot in space, including changes in its position and orientation. Subsequently, the chapter delves into the IMU measurement model, elucidating how IMUs are used to acquire dynamic information about the robot, such as acceleration and angular velocity. Furthermore, the chapter examines the position measurement model, which involves utilizing various sensor technologies to determine the robot’s specific location within its environment. Finally, the definitions of symbols used in both the conventional and proposed curvature equations, as well as parameters related to the vehicle’s path tracking model, are shown in Table 2.

### 2.1. Rigid−Body Kinematic Model

**Figure 1:**
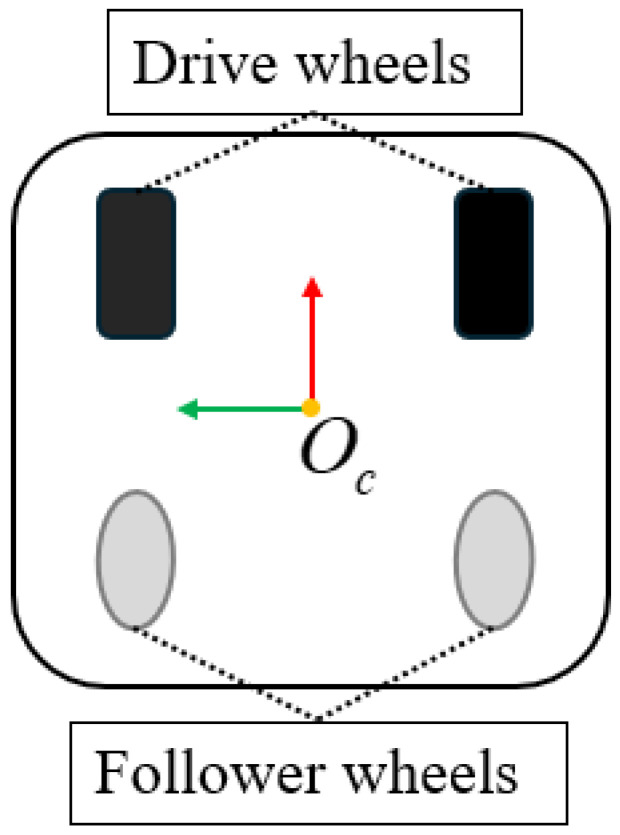
Mechanism model of a two-wheel differential robot.

Before kinematic modeling, it is necessary to agree on basic information such as the robot’s coordinate system and speed direction. The robot is considered a rigid body. We chose a simplified mechanism model to represent a two–wheel differential robot. The default agreement is shown in Figure 1. The front wheel of the robot is the driving wheel, driven by a motor, and the rear wheel is the driven wheel, which has no power and rotates with the front wheel. Different movements can be achieved by adjusting the speed of the left and right driving wheels. When the left and right driving wheels have the same speed, the robot moves in a straight line, and when the speeds are different, the robot moves in a circle.

**Figure 2:**
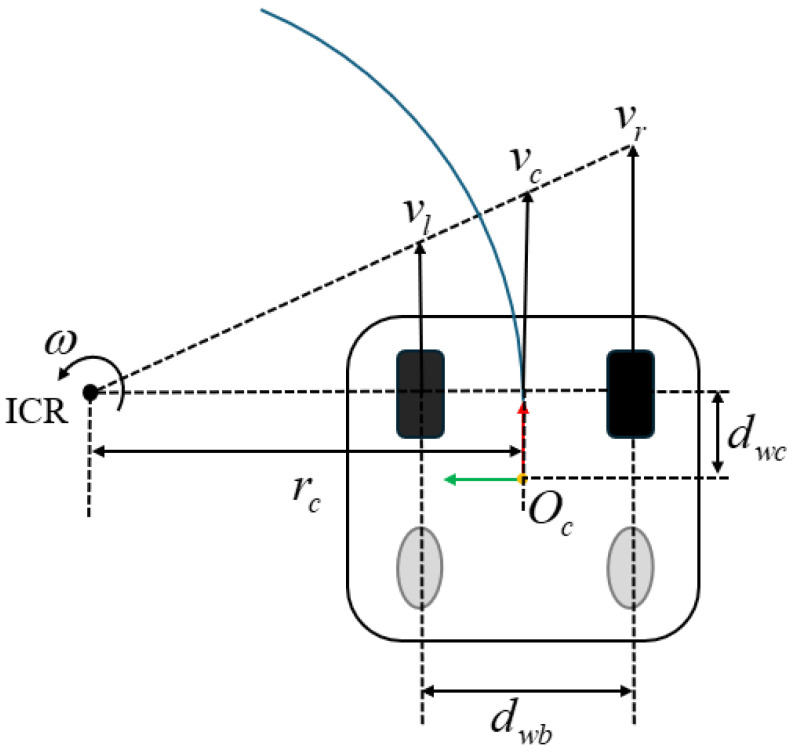
Two-wheel differential robot motion model.

All two-wheel differential robots can be simplified and represented same as the ideal model, as shown in Figure 2. In the design, the distance between the front wheel of the car and the center of the robot is $d_{wc}$. For the sake of rigor, the rotation center is marked as the center of the front wheel in the schematic diagram. The distance between the two wheels of the robot is $d_{w_{r}}$, where ICR represents the instantaneous center of rotation and the blue arcs represent the robot’s motion path. The ICR in the figure indicates that the rigid body has only rotational motion around this point, such as the rotation center axis of a gyroscope. From the theoretical concept, the robot motion can be regarded as a rigid body plane motion, so all plane rigid body motion can be decomposed into two kinds of motion: translation and rotation. Translation motion includes motion in the x direction, so the rigid body plane motion has three degrees of freedom. Constrained by the wheels, the robot’s translational motion can only move along the wheel rolling direction (x axis direction), and it cannot move along the x axis direction. Therefore, all of the points on the robot can be described using only angular velocity and the linear velocity along x. When the angular velocity is not 0, the robot is bound to be in circular motion, and, when the angular velocity is 0, the speeds of the left and right drive wheels are the same, where the robot moves in a straight line. The relationship of $v_{l}$, $v_{r}$, and $v_{c}$ can be expressed as shown in Equation (1).

(1) $$\left\{\begin{array}{cc} w & =\frac{v_{c}}{r_{c}}=\frac{v_{r}}{r_{c}+\frac{d_{wb}}{2}}=\frac{v_{l}}{r_{c}-\frac{d_{wb}}{2}}\\ v_{c} & =\frac{v_{l}+v_{r}}{2}. \end{array}\right.$$

The forward motion model of the two-wheel differential robot can be derived, that is, the motion speed of the robot body is calculated based on the speed of the left and right driving wheels, as shown in Equation (2).

(2) $$\left[\begin{array}{c} v_{c}\\ w \end{array}\right]=\left[\begin{array}{c} \frac{v_{r}+v_{l}}{2}\\ \frac{v_{r}-v_{l}}{d_{wb}} \end{array}\right]=\left[\begin{array}{cc} \frac{1}{2} & \frac{1}{2}\\ \frac{1}{d_{wb}} & -\frac{1}{d_{wb}} \end{array}\right]\left[\begin{array}{c} v_{r}\\ v_{l} \end{array}\right].$$

The two driving wheels of the differential robot are equipped with encoders, which can calculate the linear speed of the two driving wheels. Based on Equation (2), the current movement speed of the robot can be calculated and further used to calculate the robot’s odometry. The position and orientation of the robot can be updated using its motion model based on the information from the left and right wheel speeds, as shown in Equation (3).

(3) $$\left[\begin{array}{c} x_{k+1}\\ y_{k+1}\\ \theta_{k+1} \end{array}\right]=\left[\begin{array}{c} x_{k}\\ y_{k}\\ \theta_{k} \end{array}\right]+\left[\begin{array}{c} v_{c}\cdot \cos\left(\theta_{k}\right)\\ v_{c}\cdot \sin\left(\theta_{k}\right)\\ \omega \end{array}\right]\Delta t.$$

The motion state of the robot can be updated using its motion model based on the information from the left and right wheel speeds. The robot’s linear velocity $v_{c}$, angular velocity ω, and current orientation angle $\theta_{k}$ provide the necessary parameters to update the robot’s position and orientation over a defined time step Δt. Specifically, the robot’s new position, $x_{k+1}$ and $y_{k+1}$, is determined by the linear velocity, while the orientation angle $\theta_{k+1}$ is updated based on the angular velocity. This continuous process allows for effective path tracking, localization, and motion state estimation, facilitating the robot’s autonomous navigation.

### 2.2. IMU Measurement Model

IMU is a common sensor on mobile robots and mobile smart devices. Common IMUs are six-axis sensors equipped with an accelerometer that outputs three-axis acceleration and a gyroscope that outputs three-axis angular velocity. Nine-axis IMUs are also equipped with a magnetometer that outputs three-axis attitude angles. We will only discuss six-axis IMUs here. The state of IMU is usually expressed as shown in Equation  (4).

(4) XIMU=q¯TGIbgTvITGbaTpITG

where *I* represents the IMU coordinate system, and *G* represents the reference coordinate system. The attitude of the IMU is represented by rotation q¯TGI and translation pITG. More specifically, the former is the rotation that maps an arbitrary vector from *G* coordinates to *I* coordinates, which is expressed as a unit quaternion, and the latter is the three-dimensional position of the IMU under *G*. vITG represents the translational velocity of the IMU in *G*. The other two quantities, $b_{g}^{T}$ and $b_{a}^{T}$, represent the bias of the gyroscope (angular velocity meter) and accelerometer. Note the time dimension of the state quantities except the bias: the translation quantity is expressed as velocity because the IMU only provides acceleration measurement, and the rotation quantity only expresses the attitude quantity because the IMU provides angular velocity. The estimate of the state quantity can be obtained by integrating the IMU measurement.

Quaternion was used to describe the rotation quantity. The rotation variable part in the motion model can be obtained by the first-order derivative of the quaternion with respect to time. The formula for the first-order derivative of the quaternion with respect to time as shown in Equation (5).

(5) $$\dot{q}=\frac{1}{2}\left[\begin{array}{cc} \omega^{\wedge } & \omega \\ -\omega^{T} & 0 \end{array}\right]q:=\frac{1}{2}\Omega \left(\omega \right)q.$$

After sorting, the motion model equation of IMU can be obtained as shown in Equation (6).

(6) q˙′GI=12Ω(ω)q¯GIb˙g=nwgv˙IG=aGb˙a=nwap˙IG=vIG.

To mitigate the issue of error accumulation over time in the IMU, the noise and errors in the IMU model are corrected by fusing the GPS data with the Kalman filter algorithm. The IMU state is updated using the Kalman gain K, and the Kalman filter alternates between prediction and measurement updates, optimizing the state estimate by minimizing the estimation error covariance. Specifically, the fusion process involves adjusting the IMU’s predicted state using the Kalman gain to align with the GPS measurements, thereby enhancing the positioning accuracy and stability of the entire system. The error compensation and correction of the IMU is shown in Equation (7).

(7) Δϕ^IGΔv^IGΔp^IG=Kz=Kϕ^IG−ϕGPSGvIG−vGPSGpIG−pGPSG,

and the corrected state is obtained by incorporating these corrections into the IMU state estimate. The corrected state is shown in Equation (8):

(8) XIMUcorrected=XIMU−Δϕ^IGΔv^IGΔp^IG,

where the Kalman filter is employed to fuse GPS and IMU data, and the Euler angle ϕ^IG will be converted into quaternion form to ensure consistency with the state of the IMU. A detailed discussion of the Kalman filtering procedure is outside the scope of this paper, but an overview of the fusion process is provided. The Kalman gain is applied to update the IMU’s state, with the filter alternating between prediction and measurement updates. State estimation is optimized by minimizing the estimation error covariance. Specifically, the fusion process involves adjusting the IMU’s predicted state using the Kalman gain to align it with the GPS measurements, thereby improving the overall positioning accuracy and stability of the system.

## 3. Enhancements of Pure Pursuit Tracking

**Figure 3:**
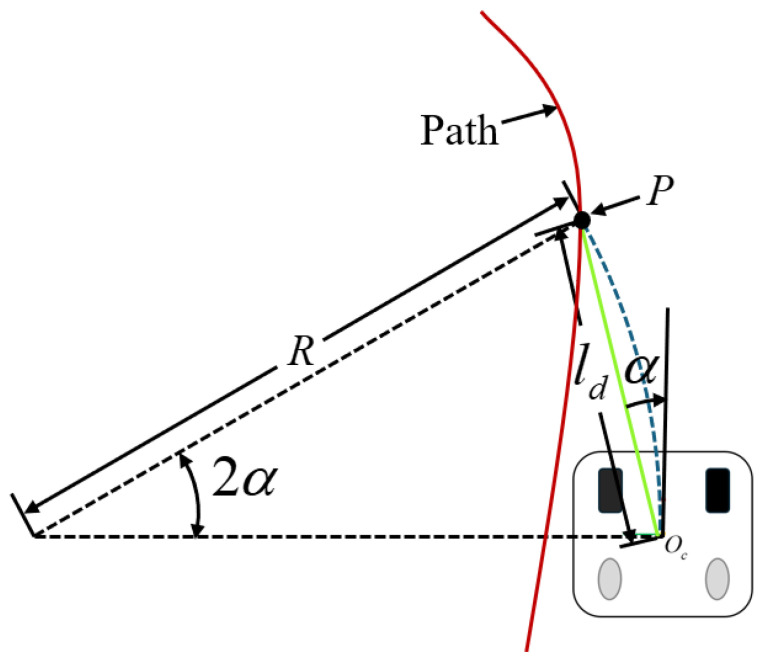
Pure pursuit geometry.

A pure pursuit principle diagram is shown in Figure 3. *P* is the next waypoint we want to track. It is located on the global path we have planned. Now, we need to control the vehicle so that the rear axle of the vehicle passes through this waypoint, where $l_{d}$ represents the distance from the current position of the vehicle (i.e., the rear axle position) to the target waypoint, and α represents the angle between the current body posture and the target waypoint. Then, according to the sine theorem, we can derive the following conversion, as shown in Equation (9):

(9) $$\frac{l_{d}}{\sin(2\alpha )}=\frac{R}{\sin\left(\frac{\pi }{2}-\alpha \right)}.$$

By sorting, we can obtain Equation (10):

(10) $$R=\frac{l_{d}}{2\sin\alpha };\kappa =\frac{2\sin(\alpha )}{\ell_{d}},$$

where κ is the curvature of the path, representing the steering angle of the two-wheel differential robot. A better understanding of this control law can be gained by defining a new variable, $e_{\ell_{d}}$, to be the lateral distance between the heading vector and the goal point, thus resulting in Equation (11):

(11) $$\sin\left(\alpha \right)=\frac{e_{\ell_{d}}}{\ell_{d}}.$$

Based on Equations (10) and (11), κ can then be rewritten as Equation (12):

(12) $$\kappa =\frac{2}{\ell_{d}^{2}}e_{\ell_{d}},$$

which shows that pure pursuit is a proportional controller of the steering angle that operates on the lateral trajectory error at some look-ahead distance in front of the vehicle with a gain of $2/\ell_{d}^{2}$. In practice, the gain is adjusted independently to maintain stability at several constant speeds, thereby specifying $\ell_{d}$ as a function of vehicle speed.

### 3.1. Proposed Model with Integral Term for Lateral Error

In the two−wheeled differential drive robot model, which consists of a front steering wheel and a rear caster wheel, the steering angle is typically calculated by assuming the robot’s steering center coincides with the front wheel center. However, since there is a distance between the front wheel center and the robot’s actual center of mass, this assumption introduces errors during turning. Specifically, when the robot attempts to turn around the front wheel center, the actual turning radius increases due to the offset between the center of mass and the steering center, resulting in a discrepancy between the required and theoretical steering angles. This discrepancy negatively impacts the accuracy of path tracking. Additionally, this offset can lead to instability, particularly when the robot turns at high speeds or in narrow spaces. Therefore, it is crucial to consider the errors induced by the center of mass offset when designing and controlling the two−wheeled differential drive robot, as well as to implement compensatory measures in both the steering angle calculations and the control system. The integral compensation formula for lateral error is shown in Equation (13).

(13) $$I\left(t\right)=\int_{0}^{t}e_{\ell_{d}}\left(\tau \right)d\tau .$$

The calculation of the integral term can be approximated using numerical integration. In discrete-time systems, the integral can be computed by accumulating the lateral error at each time step. Specifically, the integral at time step *k*, denoted as $I_{k}$, is shown in Equation (14):

(14) $$I_{k}=I_{k-1}+e_{\ell_{d}}\left(t_{k}\right)\Delta t,$$

where $I_{k}$ is the integral value at the current time step, $I_{k-1}$ is the integral value at the previous time step, $e_{\ell_{d}}\left(t_{k}\right)$ is the lateral error at the current time step, and Δt is the time step. By summing the lateral errors in this manner, the integral term incorporates the historical errors and compensates for them, thereby improving the accuracy and stability of path tracking.

Both proportional and integral terms are incorporated into the curvature equation, resulting in an updated steering angle control equation, as shown in Equation (15):

(15) $$\begin{array}{cc} \kappa (t) & =\frac{2}{\ell_{d}^{2}}\left(K_{p}\cdot e_{\ell_{d}}\left(t\right)+K_{i}\cdot I\left(t\right)\right)\\ \theta (t) & =\frac{2}{\ell_{d}^{2}}\left(K_{p}\cdot e_{\ell_{d}}\left(t\right)+K_{i}\cdot \int_{0}^{t}e_{\ell_{d}}\left(\tau \right)d\tau \right), \end{array}$$

which introduces an enhanced steering angle control law, where the integral term accounts for the accumulated error over time, providing more precise path tracking. This modification shifts the control approach from a pure proportional tracking algorithm to a proportional-integral (PI) controller. By integrating the past lateral error, the controller achieves better stability and accuracy in path tracking, especially in scenarios with significant center-of-mass offsets. The proposed control law improves the robot’s performance across varying speeds, offering enhanced robustness in both static and dynamic environments.

### 3.2. Distance Convention and Global Path Generation

When conducting an actual tracking experiment using the longitude and latitude obtained from GNSS, coordinate conversion was necessary. The global longitude and latitude coordinates provided by the GNSS were converted into the robot’s local coordinate system, which served as the basis for the tracking process.

The Haversine equation is used for calculating the great circle distance between two points on the Earth’s surface. It is based on the assumption that the Earth is a perfect sphere. We used the Haversine equation to calculate the distance between longitude and latitude and, in this way, convert the points under the LLA coordinates into points in meters, as shown in Equation (16).

(16) $$\left\{\begin{array}{c} a=\sin^{2}\left(\frac{\Delta lat}{2}\right)+\cos\left(lat_{1}\right)\cdot \cos\left(lat_{2}\right)\cdot \sin^{2}\left(\frac{\Delta lon}{2}\right)\\ c=2\cdot \mathrm{atan}2(\sqrt{a},\sqrt{1-a})\\ d=R\cdot c \end{array}\right.,$$

where $\left[lat_{1},lon_{1}\right]$ and $\left[lat_{2},lon_{2}\right]$ are the latitudes of the two locations, which are usually expressed in degrees but converted to radians in the formula. In addition, Δlat and Δlon are the differences in longitude and latitude of the two locations in radians; *a* is an auxiliary variable used to simplify the intermediate calculations in the Haversine equation, and it is used to combine the longitude and latitude differences to calculate the spherical angle distance between the two points; *c* is the spherical angle distance, which is used to represent the great circle arc degree between the two points; R means the radius of the Earth, where the unit is kilometers (equals 6378.137 km), which is used to convert spherical angle distance to actual ground distance; and d is the spherical distance between two geographical locations in the unit of the Earth’s radius r (in kilometers).

The latitude and longitude of the initial point are set as the origin, and the Haversine formula is used to calculate the distance from the origin. Then, these distances are expressed in meters, which is actually a local coordinate system centered on the origin. This coordinate system can be considered a variant of a spherical coordinate system based on the surface of the Earth.

The first step in generating a global path using the Haversine formula in conjunction with GNSS signals is to determine the latitude and longitude coordinates of the starting point. This information is provided by a GNSS receiver, which receives signals from satellites and calculates the geographic location of the receiver. Once we have the precise latitude and longitude of the starting point, we can use it as the initial point for subsequent path calculations.

As the robot moves, the GNSS system continues to provide new longitude and latitude coordinates. Using these coordinate pairs, we can apply the Haversine equation to calculate the great circle distance between any two points. By continuously calculating the distance between each point on the path and connecting these distances on a map, a global path can be generated. This path reflects the movement trajectory of the robot or vehicle relative to the surface of the Earth, providing important spatial information for navigation and route planning.

### 3.3. Coordinate Transformation

The robot has a local rectangular coordinate system, so the LLA global coordinate system (longitude, latitude, and altitude) [39] needs to be converted to the global ENU (east, north, and up) rectangular coordinate system [40]. This conversion usually needs to take into account the curvature of the Earth, but when the eccentricity e of the Earth (the ratio of the Earth’s equatorial radius to the polar radius) is very small and can be ignored, a simplified approximate formula can be used for the conversion, as shown in Equation (17).

(17) $$\left[\begin{array}{c} \Delta e\\ \Delta n\\ \Delta u \end{array}\right]=\left[\begin{array}{ccc} R\cdot \cos(lat)\cdot \Delta lon & 0 & 0\\ 0 & R\cdot \Delta lat & 0\\ 0 & 0 & \Delta alt \end{array}\right].$$

**Figure 4:**
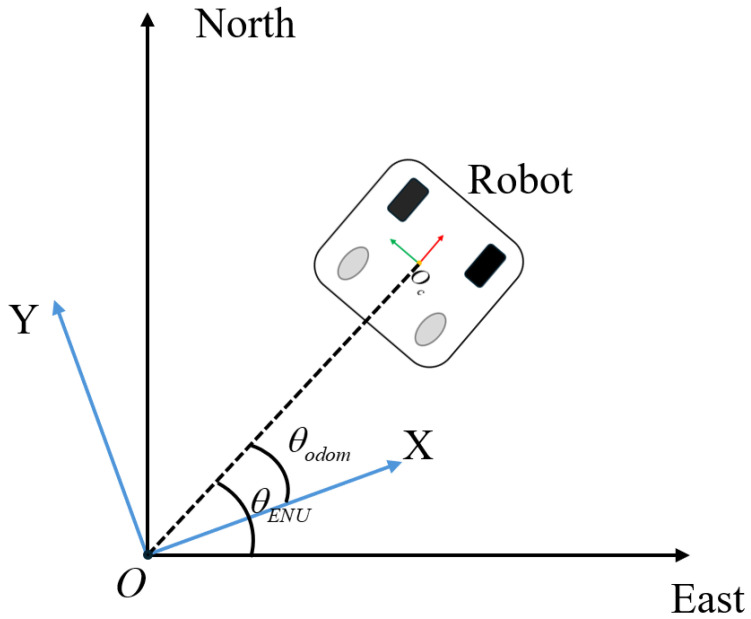
Coordinate transform relationship.

The meanings of the parameters in Equation (17) are consistent with those in Equation (16). In order to transform the points in the ENU coordinate system to the robot’s odometer coordinate system, we first need to know the robot’s yaw angle relative to the ENU coordinate system. This yaw angle is the angle between the robot’s forward direction and the geographic north. The schematic diagram of the coordinate transformation is shown in Figure 4.

The blue coordinate system is the robot odometer coordinate system, and the global coordinate system is the ENU coordinate system. It can be seen that the magnetic yaw angle is the difference between the yaw angle of the robot odometer coordinate system (odom) and the yaw angle of the ENU coordinate system, as shown in Equation (18).

(18) $$\theta =\theta_{\mathrm{odom}\;}-\theta_{\mathrm{ENU}},$$

which will be used in the rotation matrix to correctly transform the points in the ENU coordinate system to the robot’s odometry coordinate system, as shown in Equation (19). When converting LLA coordinates to ENU, the robot’s starting point is used as the origin, so the origins of the robot’s odometry coordinate system and the ENU coordinate system coincide, and no translation transformation is required.

(19) $$R_{z}\left(\theta \right)=\left[\begin{array}{ccc} \cos(\theta ) & -\sin(\theta ) & 0\\ \sin(\theta ) & \cos(\theta ) & 0\\ 0 & 0 & 1 \end{array}\right].$$

Through this rotation matrix, we can obtain the transformation relationship between the global coordinate system ENU and the robot coordinate system, as shown in Equation (20).

(20) $$\left[\begin{array}{c} X_{\mathrm{odom}\;}\\ Y_{\mathrm{odom}\;}\\ Z_{\mathrm{odom}\;} \end{array}\right]=R_{z}\left(\theta \right)\left[\begin{array}{c} E\\ N\\ U \end{array}\right].$$

### 3.4. NSGA-II Objective Function Construction

In order to improve the performance of path tracking, it is crucial to reasonably design the objective function and adjust the parameters through an effective optimization algorithm. This section will explore how to use Non−dominated Sorting Genetic Algorithm II (NGSA-II) to optimize the robot pure pursuit tracking algorithm and elaborate on the design of the objective function and its importance. This section explores the use of NGSA-II to optimize the parameters $K_{p}$ and $K_{i}$ in the robot path following control. The goal of the optimization is to improve the path tracking accuracy and the smoothness of the control input. We first defined the objective function, then described, in detail, the application process of NGSAII and the selection method of weight coefficients, and, finally, we analyzed the computational efficiency. The objective function was designed to comprehensively consider the path tracking accuracy and the smoothness of the control input. The objective function was defined as shown in Equation (21).

(21) $$F_{obj}\left(K_{p},K_{i}\right)=\left[\int_{0}^{T}\left(\omega_{1}\cdot e_{\ell d}{\left(t\right)}^{2}\right)dt,\int_{0}^{T}\left(\omega_{2}\cdot u{\left(t\right)}^{2}\right)dt\right],$$

where the first part calculates the sum of squares of the lateral error $e_{\ell d}\left(t\right)$, and the goal is to minimize the error to improve the path tracking accuracy. The second part calculates the sum of squares of the control input u(t), and the goal is to reduce the volatility of the input signal and thus improve the smoothness of the control input. Here, u(t) is the steering angle θ(t), as shown in Equation (15). The weight coefficients $\omega_{1}$ and $\omega_{2}$ are used to balance the relative importance of these two objectives. The square of the error is used as part of the objective function because it is mathematically continuous and differentiable, making the optimization problem easier to handle. The square of the error also has the effect of treating negative errors and positive errors equally, avoiding the problem of positive and negative errors canceling each other out.

### 3.5. Parameter Optimization and Logic Using NSGA-II Algorithm

In the optimization of the objective function using the NSGA-II algorithm, the process begins with population initialization, where a set of random control parameter solutions, specifically the initial values of $K_{p}$ and $K_{i}$, are generated. After the objective function is established, parameter optimization is performed using the NSGA-II algorithm. The fitness of each individual is evaluated by calculating the objective function value, which is based on non-dominated sorting. Following this, individuals with higher fitness are selected for crossover and mutation, generating a new generation of individuals. The population is updated through non-dominated sorting and crowding distance ranking, iterating until the stopping criteria, such as a maximum number of iterations or the achievement of the optimization goal, is achieved.

Parameter optimization is a crucial step in enhancing the performance of the path tracking system. The goal is to find a balance in a multi−objective optimization framework that minimizes path error and improves control accuracy and stability while also considering computational efficiency. As a powerful multi−objective optimization algorithm, NSGA-II can effectively balance multiple objectives and find the optimal control parameters. Specifically, the algorithm searches the parameter space through genetic operations (selection, crossover, and mutation), ensuring performance improvements while avoiding overfitting or local optima.

To further assess the optimization results, the NSGA-II algorithm uses non-dominated sorting and crowding distance ranking to select a set of superior solutions, which are further explored through crossover and mutation operations to find potentially better solutions. The optimization result is typically evaluated by minimizing path tracking error, which measures the deviation of the robot from the target trajectory. Minimizing this error improves path tracking precision, ensuring that the robot follows the planned path as accurately as possible.

Therefore, by minimizing tracking error, the NSGA-II algorithm selects control parameters with better path tracking performance, with the optimization result verified through the calculation of path errors and the effectiveness of control inputs.

In the final step, the algorithm outputs the optimal control parameters, which represent the best control strategy for the current path following task. The optimization results are validated by verifying that the selected parameters can achieve the desired navigation performance in real world tasks. By minimizing path error, the NSGA-II algorithm not only optimizes the control parameters, but also enhances the precision and stability of path tracking, ensuring that the robot can maintain good performance under varying speeds and environmental conditions. The pseudo code of the overall process is shown in Algorithm 1.


**Algorithm** **1** NSGA-II Algorithm Optimization for Path Following Control
  1:
**Input:**
  2:   Initial population size, mutation probability, stopping criteria  3:
**Output:**
  4: Optimal values for $K_{p}$ and $K_{i}$  5:**Initialize** population with random $K_{p}$ and $K_{i}$  6:**while** stopping criteria are not met **do**  7: **Evaluate** fitness for each individual  8: **for** each individual **do**  9:  Simulate path following with $K_{p}$ and $K_{i}$10:  Compute fitness based on the objective function11: **end for**12: **Select** parents based on fitness13: **Crossover** to generate offspring14: **for** each parent pair **do**15:  Perform crossover to create new individuals16: **end for**17: **Mutate** offspring18: **for** each offspring **do**19:  **if** mutation probability is met **then**20:    Adjust $K_{p}$ or $K_{i}$21:  **end if**22: **end for**23: **Evaluate** fitness for offspring24: **for** each offspring **do**25:  Simulate and compute fitness26: **end for**27: **Update** population28: Replace old population with offspring or a mix of both29:
**end while**
30:**Return** the best individual as the optimal solution



## 4. Results

### 4.1. Robot Platform

For actual application, we chose an outdoor campus environment as the test scenario for algorithm evaluation. In order to ensure accurate evaluation of the robot’s walking path, we selected a high-precision GNSS system to obtain the true value data of the path. In the following section, we will introduce the initialization process of GNSS high-precision positioning in detail to ensure the accuracy and reliability of positioning data and provide a solid foundation for subsequent evaluation of the algorithm.

**Figure 5:**
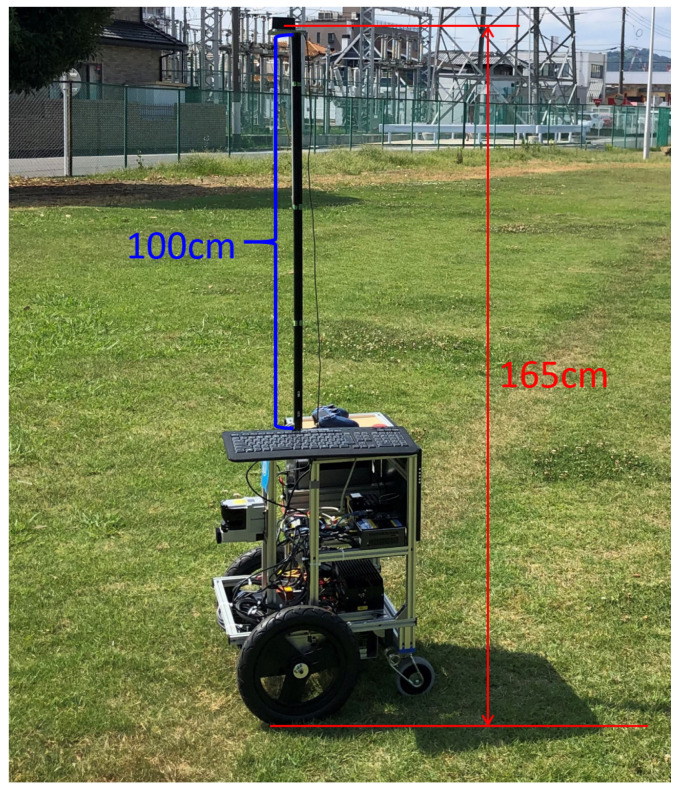
GNSS antenna installation diagram.

In this experiment, an AQLOC device was used, which is a quasi-zenith satellite system manufactured by Mitsubishi Electric. It is a high-precision positioning terminal that supports the CLAS (Centimeter Level Augmentation Service). In the positioning accuracy experiment, we used a high-precision time series data collection method. By continuously observing the device in a stationary state, we recorded the error values of the longitude and latitude coordinates. During the experiment, we ensured the stability of the measurement equipment and the consistency of environmental conditions to reduce the impact of external factors on the measurement results. With this method, we can accurately evaluate the accuracy performance of the positioning system in different coordinate directions, thereby verifying its reliability and accuracy in practical applications. For the installation of the GNSS antenna, we used a 100 cm high 5-series MISUMI aluminum frame, which is fixed to the robot body at two angles. The actual state of the experiment is shown in Figure 5.

The serial communication tool uses Linux OS Putty software, as shown below. The data collection uses Putty’s log function to collect GNSS raw data from the serial port. The GNSS device and the Linux machine are connected via a USB cable. The serial communication specifications are as follows:

- Baud rate: 115, 200.
- Data: 8 bit.
- Parity: none.
- Stop: 1 bit.
- Flow control: none.

### 4.2. GNSS Calibration

Our robot was placed outdoors and remained stationary, as shown in the right figure. The test results are shown in the table and figures. The table shows the range, minimum, maximum, and average values of the GNSS coordinates. The data in the table are very small, which verifies that the GNSS signal has almost no drift and proves that GNSS has good positioning accuracy. GNSS high-precision positioning has high accuracy in a stationary state, as shown in Table 3. Specifically, the longitude error range is between [−0.027 m, 0.029 m], the minimum error is −0.027 m, the maximum error is 0.029 m, and the average error is only −0.012 m. The latitude error range is between [−0.019 m, 0.014 m], the minimum error is −0.019 m, the maximum error is 0.014 mm, and the average error is 0.011 m. These data show that the GNSS high−precision positioning system has a small error range when positioning and can provide very accurate position data for the robot.

**Figure 6:**
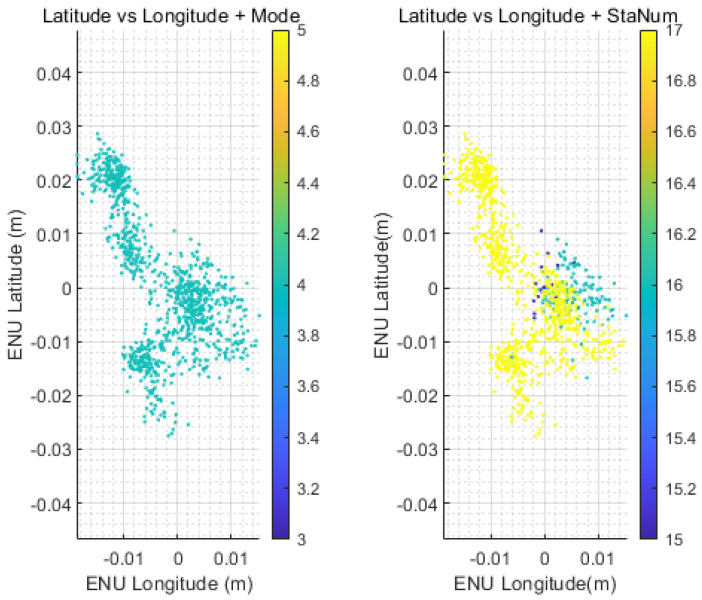
GNSS positioning mode and satellite numbers during measurement.

The relationship between the measurement mode and satellite numbers with GNSS points is shown in Figure 6. All of the data points are in Measurement Mode 4, i.e., measured in RTK Fix Mode, which typically indicates very stable signal reception and high-precision operation. The right figure displays the relationship between our measurement results and the number of satellites, which remained between 15 and 17, thus providing accurate position measurements.

### 4.3. Path Generation

**Figure 7:**
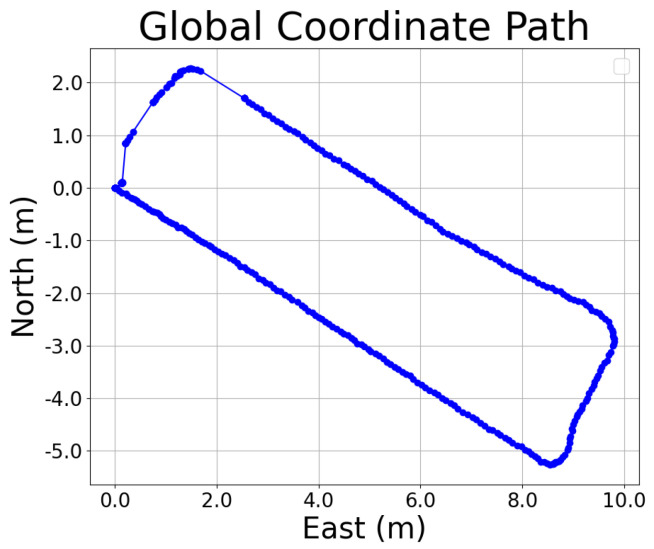
ENU coordinate path.

**Figure 8:**
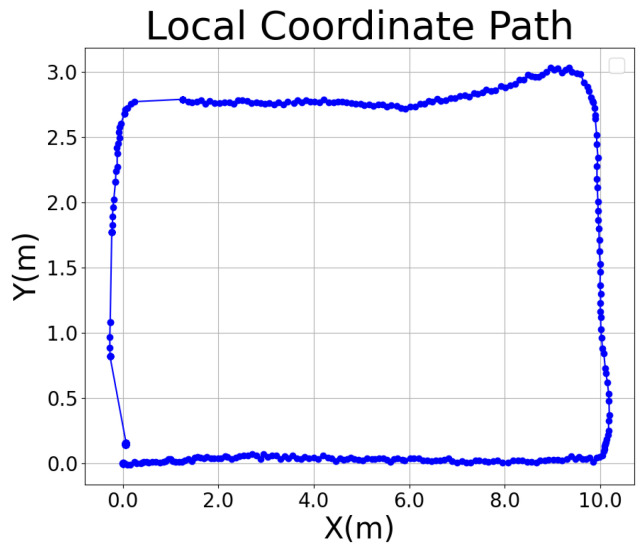
Robot coordinate path.

After acquiring the raw GNSS data, the raw GNSS coordinates are converted into a global path represented in the East-North-Up (ENU) coordinate system, as shown in Figure 7. Subsequently, the global coordinate system is transformed into a local coordinate system suitable for path tracking by calculating the deviation between the heading angle provided by the IMU sensor and the geographic heading angle derived from GNSS, as shown in Figure 8, which ensures the accuracy and consistency of the coordinate system conversion, providing a precise reference for the implementation of the subsequent path tracking algorithms.

### 4.4. Path Following Result Analysis

This section aims to evaluate the performance of the NSGA-II optimized path tracking system by performing a series of tests to ensure that the system meets the predetermined performance criteria. The tests include path tracking accuracy, the efficiency of the optimization algorithm, and the stability of the entire system. In order to comprehensively evaluate the system performance, we defined the following key performance indicators (KPIs): path tracking average absolute pose error, total tracking error control parameters (Kp and Ki) maximum linear velocity look ahead distance, and the evolution of the optimal value of the fitness function over generations. The performance of the algorithm was tested by combining different look ahead distances and maximum linear velocities. The results are shown in Table 4.

According to the data, the optimized algorithm showed higher accuracy than the original algorithm in all test scenarios. Specifically, the average error of the optimized algorithm was significantly lower than that of the original algorithm, whether at smaller maximum linear speeds (such as 1 and 3) or larger maximum linear speeds (such as 4 and 5). When the maximum linear speed was 5 and look ahead distance was 0.5, the average error of the original algorithm was 0.5175, while the average error after optimization was reduced to 0.191183, showing the effectiveness of optimization measures in reducing path tracking errors.

**Figure 9:**
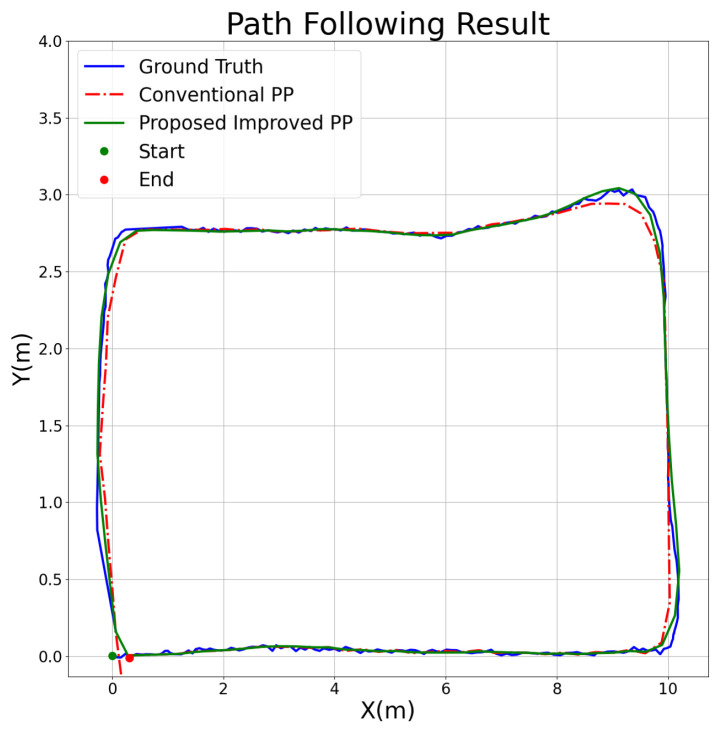
Path following comparison under look ahead distance = 0.5 and max linear speed = 3.

**Figure 10:**
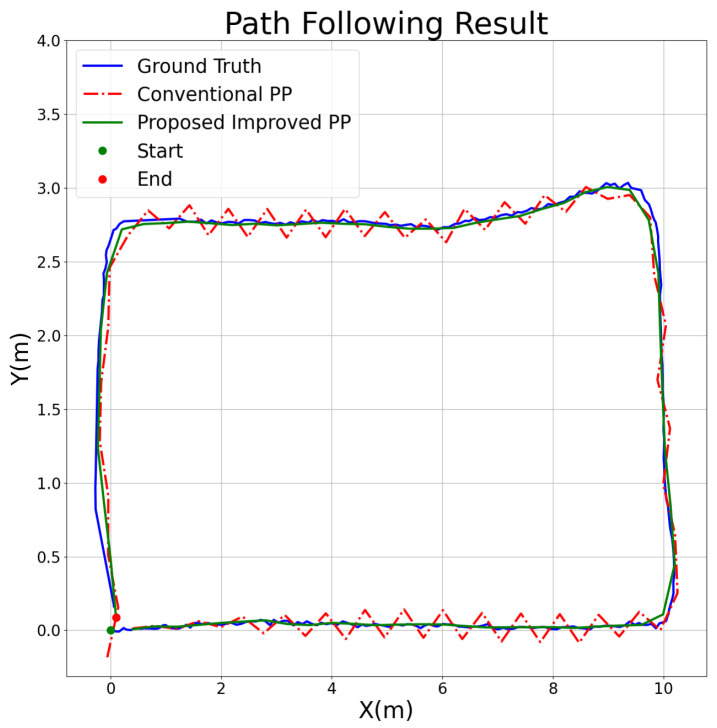
Path following comparison under look ahead distance = 0.5 and max linear speed = 4.

The optimized results have a smaller APE value. As such, after optimization, the overall error had significantly improved. For a better understanding, we analyzed two different scenarios: first is the conventional algorithm, which does not exhibit oscillations, whereas oscillations are presented in the second one. We compared and illustrated the path tracing results for a current view distance of 0.5 and a maximum linear speed of 3, as shown in Figure 9, as well as under a linear speed of 4, as shown in Figure 10.

The results clearly demonstrate the significant advantages of the proposed improved pure pursuit algorithm under various experimental conditions. We selected specific scenarios to illustrate the conventional algorithm’s performance in both stable and potentially unstable conditions. In one case, where the look ahead distance was set to 0.5 m and the maximum linear velocity was 3 m/s, the proposed algorithm achieved more accurate tracking of the ground truth path (blue line) compared to the conventional pure pursuit method (red dashed line), with significantly reduced tracking deviations. In this scenario, the absolute pose error (APE) was reduced by 14.633%, indicating that the improved algorithm enhances tracking precision under moderate speed conditions. Additionally, under higher-speed conditions (look ahead distance = 0.5 m and maximum speed = 4 m/s), the conventional algorithm exhibited increased tracking errors and instability, particularly in curved sections of the path, while the proposed algorithm maintained greater stability, achieving a 55.944% reduction in APE. This performance improvement was consistent across other tested conditions, showing that the proposed algorithm provides substantial enhancements not only in low speed conditions, but also in higher speed scenarios. Thus, the proposed algorithm demonstrates significant advantages over the conventional method in terms of accuracy and stability, regardless of the specific parameter settings.

### 4.5. NSGA-II Iteration Result Analysis

**Figure 11:**
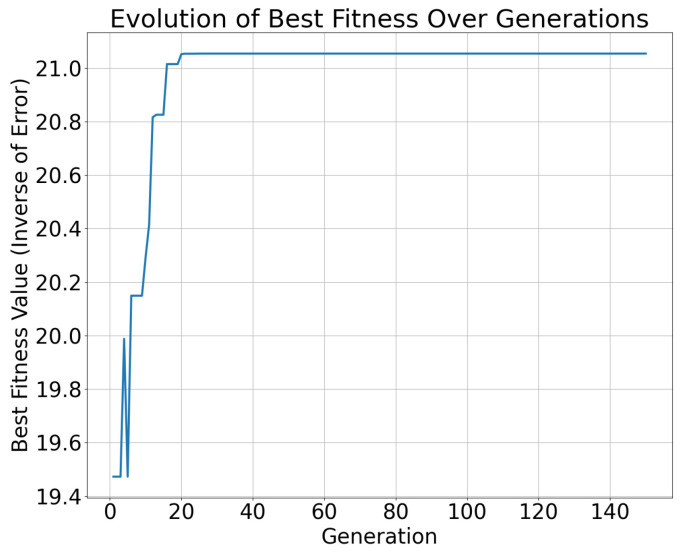
Evolution of best fitness under look ahead distance = 0.5 and max linear speed = 3.

**Figure 12:**
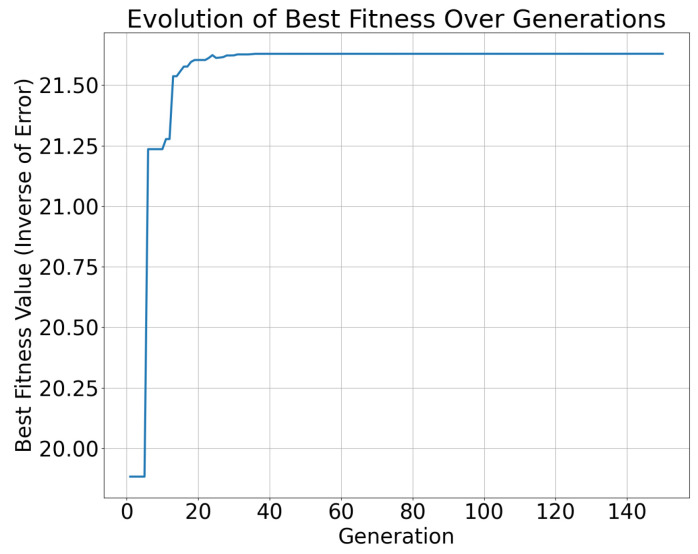
Evolution of best fitness under look ahead distance = 0.5 and max linear speed = 4.

In the optimization process of the NSGA-II, the best fitness value showed a gradually increasing trend with the number of iterations, indicating that the algorithm was progressively approaching a better solution. Figure 11 and Figure 12 illustrate this trend, where the horizontal axis represents the number of iterations and the vertical axis represents the best fitness value, which can be interpreted as the inverse of the error. From the experimental results, it was observed that, as the iterations progressed, the fitness value of the best individual increased with each generation, indicating that the algorithm was optimizing the path tracking control parameters and gradually reducing the path tracking error.

Additionally, different control parameters, such as the proportional gain $K_{p}$ and integral gain $K_{i}$, had a significant impact on tracking accuracy. Experimental results demonstrate that appropriate values of $K_{p}$ and $K_{i}$ can significantly improve the accuracy and stability of path tracking. However, excessively high or low values may lead to either an overly fast or slow system response, affecting the path tracking performance. Therefore, optimizing these parameters is crucial for improving the overall performance of robot navigation and path tracking systems.

Compared to other optimization algorithms, the advantage of NSGA-II lies in its ability to simultaneously consider multiple factors for global search, thus avoiding the trap of local optima. In contrast, some gradient based methods, such as gradient descent, may have an advantage in convergence speed but are prone to becoming stuck in local optima and cannot handle multi objective optimization problems. Therefore, NSGA-II offers unparalleled advantages in global optimization, avoiding local optima and multi objective optimization.

### 4.6. Real Time Performance Test and Analysis

**Figure 13:**
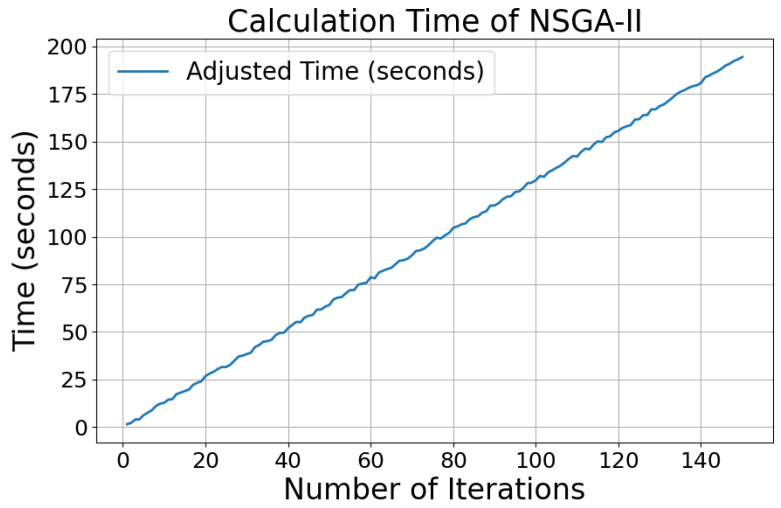
Calculation time in different iteration numbers of NSGA-II.

The calculation time of the NSGA-II algorithm was evaluated across various iteration counts, and the results are presented in Figure 13. It is evident that the computation time increases linearly with the number of iterations. Based on the experimental data, full convergence of the algorithm was achieved after 40 generations, with a convergence time of 52.287 s, as illustrated in Figure 11 and Figure 12.

During the robot initialization phase, in order to complete the initial positioning, the robot remains stationary for 1 to 2 min. This waiting period allows sufficient time for the computation of the proportional control parameter ($K_{p}$) and the integral control parameter ($K_{i}$), as well as for the construction of the path tracking logic and the initial GNSS positioning.

Additionally, the data processing pipeline was optimized to minimize delays, ensuring that real time performance is maintained. The robot platform, based on the Robot Operating System (ROS), incorporates an efficient data processing mechanism, ensuring that, even in the presence of data delays, tracking performance and accuracy are not significantly impacted.

From the analysis, it can be concluded that the current implementation of the NSGA-II algorithm not only meets real time performance requirements, but also effectively supports a robot’s navigation and path tracking tasks. The optimized processing mechanisms ensure the system operates within acceptable real time constraints, validating its feasibility for real world applications.

## 5. Conclusions

In this work, we designed and implemented an advanced navigation system utilizing an improved pure pursuit path tracking algorithm that is specifically tailored for paths generated from GNSS signals. The primary objective was to enhance the system’s path tracking performance, which was achieved by optimizing control parameters using the NSGA-II algorithm. This approach led to significant improvements in tracking accuracy compared to conventional methods, making the system more reliable for real world applications.

A critical improvement was made to the pure pursuit algorithm’s turning performance by introducing a proportional integral (PI) factor, which allowed for more accurate and smoother turns while following the GNSS derived trajectory. The appropriate controller gains were determined through the NSGA-II iterations, ensuring that the system could adapt to changes in speed and curvature along the path. To validate the effectiveness of the proposed algorithm, extensive comparative experimental results were conducted, demonstrating that our algorithm outperforms traditional path tracking methods in terms of both tracking accuracy and stability. Specifically, the improved algorithm exhibited reduced tracking errors and provided a more robust response when following paths generated from GNSS data, which are often prone to noise and inaccuracies.

In conclusion, the enhanced pure pursuit algorithm, optimized by NSGA-II, shows significant improvements over the traditional pure pursuit tracking method, enhancing tracking stability and reliability in practical navigation tasks, and its applicability in mobile robots equipped with high precision GNSS navigation was also validated.

## 6. Future Work

We plan to further investigate the robustness of the NSGA-II optimized parameters across different environments and task scenarios. While the current study focuses on optimizing path following control parameters under specific conditions, we aim to explore how these optimized parameters perform in varying environments, such as urban areas, forests, or indoor spaces with complex obstacles. Additionally, the performance of the algorithm in dynamic environments, where the robot’s operating conditions may change during navigation, will be thoroughly evaluated.

We also plan to extend the current study by incorporating comparative analyses with other widely used path tracking algorithms, such as Model Predictive Control (MPC) and Stanley controllers. These comparisons will allow us to better evaluate the performance of our enhanced pure pursuit path tracking algorithm in terms of accuracy, stability, and computational efficiency. By implementing and testing these methods under identical conditions, we aim to highlight the advantages and potential limitations of our approach. Additionally, we intend to explore hybrid algorithms that integrate the strengths of multiple techniques to further optimize path tracking performance, especially in complex and dynamic environments.

## Figures and Tables

**Table 1:** The advantages and limitations of path tracking algorithms.

Method	Advantages	Limitations
Pure Pursuit	Simple implementation; works well at low speeds.	Poor accuracy at high speeds; struggles with sharp turns and complex paths.
Model Predictive Control (MPC)	Provides optimal control by considering future states; adaptable to complex paths.	High computational cost; requires accurate models; limited real−time performance.
Multi−Sensor Fusion	Enhances robustness by combining multiple data sources; improves accuracy.	High complexity in data fusion; resource−intensive; potential sensor data inconsistencies.
Visual Tracking	Capable of recognizing and tracking dynamic obstacles; rich environmental information.	Performance affected by lighting changes and occlusions; requires high processing power.

**Table 2:** Definition of the parameters in Section 3.

Parameter	Description
$v_{c}$	The linear velocity of the robot
ω	The angular velocity of the robot
$v_{r}$	The speed of the right driving wheel
$v_{l}$	The speed of the left driving wheel
$d_{wb}$	Wheelbase distance
$d_{wc}$	The perpendicular distance to ICR
$r_{c}$	The radius of rotation
ICR	The center of rotation
*P*	Target point on the path
$o_{c}$	Center of the vehicle
*R*	Radius of the arc
2α	Angle of the arc
α	Angle between current posture and *P*
$l_{d}$	Look ahead distance
κ	The curvature of the arc
$e_{l_{d}}$	Horizontal lateral error to *P*
θ(t)	Heading angle adjustment function
$K_{p}$	Proportional gain
$K_{i}$	Integral gain
I(t)	Integral of lateral error over time
$P_{k_{p},k_{i}}$	Population of parameter pairs $\left(K_{p},K_{i}\right)$
$e_{\mathrm{avg}}$	Average path following error
*N*	Number of data points
$x_{i},y_{i}$	Actual position coordinates of the *i*-th point
$x_{gt,i},y_{gt,i}$	Goal position coordinates of the *i*-th point
ϵ	Positive value added for numerical stability
$f(K_{p},K_{i})$	Fitness function of $K_{p}$, $K_{i}$

**Table 3:** Positioning accuracy results in a stationary state based on time series data.

Coordinate	Range	Min	Max	Average
Longitude	[−0.027 m, 0.029 m]	−0.027 m	0.029 m	−0.012 m
Latitude	[−0.019 m, 0.014 m]	−0.019 m	0.014 m	0.011 m

**Table 4:** System performance evaluation.

Look Ahead Distance	Max Linear Speed	Average Absolute Pose Error (APE)		Best Kp	Best Ki
		Conventional PP	Proposed Enhanced PP		
0.5	3	0.056	**0.047**	0.6283	0.0
0.5	4	0.105	**0.046**	0.5857	0.0072
0.5	5	0.518	**0.191**	0.7052	0.3674
1	4	3.548	**0.067**	0.6896	0.0
1	5	42.071	**0.064**	0.7422	0.2001
1.5	4	0.098	**0.089**	0.8852	0.0
1.5	5	3.425	**0.087**	0.836	0.3015

## Data Availability

The data that support the findings of this study are available from the author, Seiji Hashimoto, upon reasonable request.

## Abbreviations

The following abbreviations are used in this manuscript:

GNSS	Global Navigation Satellite System
INS	Inertial Navigation System
RTK	Real Time Kinematic
IMU	Inertial Measurement Unit
ODO	Odometer
LPS	Localization Positioning System
PI	Proportional Integral (Control)
APE	Absolute Pose Error
NSGA-II	Non−dominated Sorting Genetic Algorithm II
ENU	East−North−Up (Coordinate System)
LLA	Longitude, Latitude, Altitude (Coordinate System)
